# Storing single photons emitted by a quantum memory on a highly excited Rydberg state

**DOI:** 10.1038/ncomms14072

**Published:** 2017-01-19

**Authors:** Emanuele Distante, Pau Farrera, Auxiliadora Padrón-Brito, David Paredes-Barato, Georg Heinze, Hugues de Riedmatten

**Affiliations:** 1 ICFO-Institut de Ciencies Fotoniques, The Barcelona Institute of Science and Technology, Castelldefels, 08860 Barcelona, Spain; 2 ICREA-Institució Catalana de Recerca i Estudis Avançats, 08015 Barcelona, Spain

## Abstract

Strong interaction between two single photons is a long standing and important goal in quantum photonics. This would enable a new regime of nonlinear optics and unlock several applications in quantum information science, including photonic quantum gates and deterministic Bell-state measurements. In the context of quantum networks, it would be important to achieve interactions between single photons from independent photon pairs storable in quantum memories. So far, most experiments showing nonlinearities at the single-photon level have used weak classical input light. Here we demonstrate the storage and retrieval of a paired single photon emitted by an ensemble quantum memory in a strongly nonlinear medium based on highly excited Rydberg atoms. We show that nonclassical correlations between the two photons persist after retrieval from the Rydberg ensemble. Our result is an important step towards deterministic photon–photon interactions, and may enable deterministic Bell-state measurements with multimode quantum memories.

## Abstract (web summary)

The state of a single photon can be stored as a Rydberg excitation using electromagnetically induced transparency, and this enables nonlinear interactions at the single-photon level. Here, the authors store a paired photon emitted by a quantum memory in an ensemble-based, highly nonlinear medium.

Efficient photon–photon interactions require a highly nonlinear medium that strongly couples with a light field, a single-photon source compatible with the medium and the ability to coherently map the photon in and out of the nonlinear medium^1^. In addition, for quantum repeaters applications for long-distance quantum communication, the single photon should be part of a correlated photon pair generated by a quantum memory (QM), which allows for synchronization along the communication line^2^. Nonlinearity at the single-photon level has been demonstrated with a variety of systems, including single atoms and atomic ensembles^3^,^4^,^5^,^6^,^7^,^8^,^9^,^10^,^11^,^12^,^13^ as well as nonlinear crystals albeit with small efficiency^14^. However, the coupling of true single photons with a highly nonlinear medium has been demonstrated so far only with single atoms^11^,^12^. These systems are inherently nonlinear but suffer from low light–matter coupling in free-space and therefore require experimentally challenging high-finesse cavities.

Using highly excited Rydberg states of atomic ensembles can be a simpler alternative. The atomic ensemble ensures a strong light–matter coupling and the dipole–dipole interactions between Rydberg states enable strong, tunable nonlinearities. For a sufficiently dense ensemble 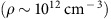 and at sufficiently high quantum number of the Rydberg state (*n*≥60), nonlinear response at the single-photon level has been already demonstrated^15^,^16^,^17^,^18^,^19^,^20^ and has been exploited to implement a number of operations with weak coherent states (WCSs)^21^,^22^,^23^,^24^. Entanglement between a light field and a highly excited Rydberg state^25^ has also been recently demonstrated.

While single-photon nonlinearities have been demonstrated with WCSs, efficient quantum information processing using this system requires two additional steps. First, a single-photon source that matches the frequency and the sub-MHz spectral bandwidth of the Rydberg excitation, and, second, the ability to store and retrieve the input single photon. The latter is of key importance for implementing high-fidelity photonic quantum operations using excited Rydberg states^23^,^26^,^27^, and, in addition, it has been shown to be beneficial to enhance the nonlinear response of this kind of systems^28^. While storage and retrieval of a single photon transmitted between remote atomic ensembles has been achieved in ground states^29^,^30^,^31^,^32^,^33^ or low-lying Rydberg states^34^, storing it in a highly nonlinear Rydberg ensemble presents additional experimental challenges, such as high sensitivity to stray fields, stronger motional-induced dephasing due to the large wavelength mismatch between the single photon and the coupling laser, weak oscillator strength requiring higher intensity of the coupling beam, as well as strong focusing of the single-photon needed to achieve nonlinearity at low light power. These challenges make it more difficult to achieve the required signal-to-noise ratio (SNR) to preserve the quantum character of the stored and retrieved field.

Here we demonstrate storage and retrieval of a paired and synchronizable single photon in a highly nonlinear medium based on excited Rydberg atomic states of a cold atomic ensemble. This is realized by using a photon source based on a read-only cold atomic ensemble QM^35^ with which we can generate pairs of non-classically correlated photons that fulfil the frequency and the narrow bandwidth requirement of the Rydberg medium. In the generation stage (site A in Fig. 1), after a successful heralding event a single photon is emitted at a programmable delay time *t*_A_ allowing for potential synchronization between different pair sources. The photon is then collected into an optical fibre and sent to a remote atomic ensemble (site B in Fig. 1) where it is stored as a collective Rydberg excitation and retrieved after a storage time *t*_B_ The storage and retrieval in high-lying Rydberg states is realized with sufficiently high SNR (>20) to enable the demonstration of highly non-classical correlations between the heralding photon and the highly excited Rydberg collective excitation, and preservation of the single-photon character of the retrieved field. Finally, we also demonstrate the highly nonlinear response of our medium with WCSs containing tens of photons. The last result is obtained in a cloud with moderate density 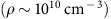 and can be easily improved to reach single-photon nonlinearity via well-known atom trapping techniques. Combining a source of narrow-band correlated single photons with a highly nonlinear medium at high SNR, our system is a building block for future quantum networks with deterministic operations.

## Results

### Experimental set-up

**Figure 1:**
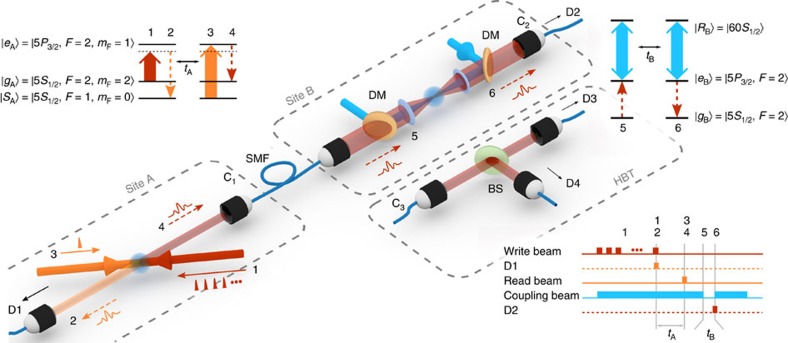
Experimental set-up with relevant atomic transitions and pulse sequence. Following the numbering in the pulse sequence, in site A, we (1) send a series of write pulses (red solid arrow), (2) probabilistically detect a write photon (orange dashed line) by single-photon detector (SPD)) D1, (3) send an intense read pulse (orange solid arrow) after a storage time *t*_A_ generating deterministically (4) a read photon (red dashed line) that is sent to site B through a single-mode fibre (SMF). In site B, a counterpropagating, coupling beam (blue arrow) converts the read photon into a slowly propagating dark-state polariton. Here we (5) switch-off the coupling beam, storing the read photon and (6) switch it on again after a storage time *t*_B_ retrieving the photon that is detected by SPD D2. The coupling beam and the read photon are both focused by the same pair of aspheric lenses and are combined and separated by dichroic mirrors (DMs). Hanbury Brown–Twiss (HBT) set-up is shown in another box. The field to be analysed emerges from the SMF at position C_3_, it is split by a 50:50 beam splitter (BS) and detected by two detectors, D3 and D4 afterwards. To analyse the photon statistic before and after storage in the Rydberg state, we connect the HBT set-up either at position C_1_ or at C_2_.

A schematic of the experiment is shown in Fig. 1. In site A, we implement a photon-pair source with controllable delay, using a cold atomic QM based on the Duan–Lukin–Cirac–Zoller (DLCZ) scheme^35^,^36^. We use a cold atomic ensemble of ^87^Rb atoms. Atoms initially prepared in the ground state 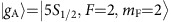 are illuminated with a series of weak coherent pulses at 780 nm (write pulses) red detuned by Δ=40 MHz with respect to the 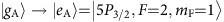 transition so that a write photon is probabilistically created via Raman scattering and detected by single-photon detector (SPD) D1. This heralds a single collective excitation in the state 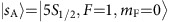 (Supplementary Note 1). The excitation can be deterministically readout after a controllable storage time *t*_A_ by means of a strong, counterpropagating read pulse on resonance with the 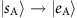 transition. The read pulse creates a 350 ns-long (full width at half maximum, FWHM) read photon in a well-defined spatiotemporal mode resonant with the 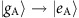 transition. The read photon is collected and sent through a 10 m single-mode fibre to site B, where a separate ensemble of cold ^87^Rb atoms is prepared in the state 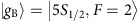. We estimate that the probability to obtain a single photon in front of ensemble B conditioned on the detection of a write photon (the heralding efficiency) is *η*_H_=0.27. At site B, a coupling beam at 480 nm resonant with the 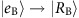, transition creates the condition for electromagnetically induced transparency (EIT)^37^,^38^,^39^ (Supplementary Note 3; Supplementary Fig. 2), where 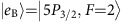 and 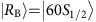. This converts the read photon into a slow-propagating Rydberg dark-state polariton (see Supplementary Note 4). By adiabatically switching off the coupling beam, the read photon is stored as single collective Rydberg atomic excitation^40^ and the state of the ensemble reads:


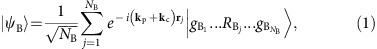


where *N*_B_ is the number of atoms in the interaction region, and **k**_p_ and **k**_c_ the wavevector of the single photon and coupling beam, respectively. The stored excitation is retrieved after a storage time *t*_B_ by switching the coupling beam back on and detected by a SPD D2 (Supplementary Fig. 3). The read photon waveform in ensemble A can be tailored by shaping the read pulse^41^ to maximize the SNR of the storage in site B. Notice that to match the frequency of the single photon emitted at site A, we have to employ at site B the 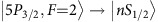 instead of the most commonly used and stronger 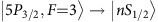 transition^15^,^18^,^19^,^23^,^24^. This makes it more challenging to reach high storage efficiency of the single photon into the collective Rydberg state.

### DLCZ QM

**Figure 2:**
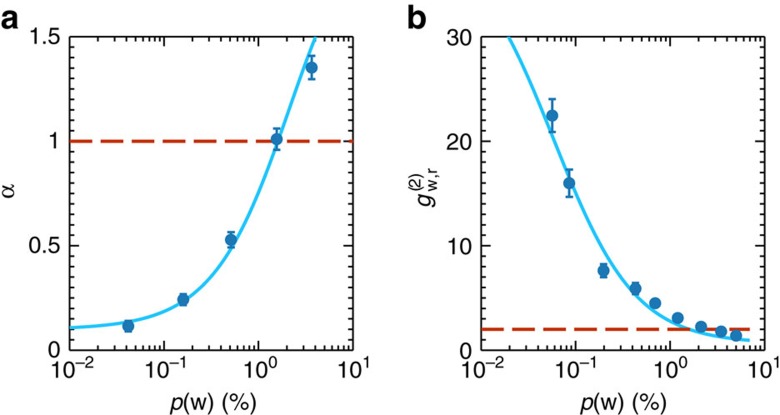
Anti-bunching parameter and cross-correlation function 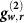 without storage in site B. (**a**) Anti-bunching parameter *α* measured before site B and (**b**) cross-correlation function 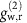 measured after site B without loading the atomic ensemble. Data are taken at *t*_A_∼1 μs. For low *p*(w) high-quality heralded single photon in the read mode as well as non-classical correlations are created, beating the classical bounds (indicated by the dashed line). The solid lines are fits with a model described in Supplementary Note 2. The error bars are the propagated Poissonian error of the photon counting probabilities.

First, at fixed *t*_A_∼1 μs, we characterize the DLCZ memory in site A as a source of high-quality synchronizable single photons when no storage in site B is performed, as shown in Fig. 2. The read single-photon quality is inferred by measuring its heralded anti-bunching parameter *α*=*p*(r_3_, r_4_|w)/*p*(r_3_|w)*p*(r_4_|w) via Hanbury Brown–Twiss (HBT) measurement before the 10 m single-mode fibre. We also measure the second-order, cross-correlation function 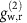
=*p*_0_(w, r_2_)/*p*(w)*p*_0_(r_2_) of the paired write and read photons without loading the atoms in site B. Here *p*(w) (*p*(r_*i*_)) is the probability to detect a write (read) photon by SPD D1 (D*i*, with i=2, 3, 4), while *p*(*x*, *y*) is the probability of coincident detection event *x* and *y* and *p*(*x*|*y*) is the conditional probability of event *x* conditioned on *y*. The subscript 0 indicates that no atoms are loaded in site B. At low *p*(w), a successful detection of a write photon projects the read mode into a high-quality single-photon state, with measured values as low as *α*=0.11±0.02 at *p*(w)=0.04%, shown in Fig. 2a. In the same condition strong non-classical correlations are found, 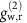 being well above the classical bound of two for a state emitted by a DLCZ QM (assuming thermal statistics for the write and read fields, see Supplementary Note 1). At higher *p*(w), multiple excitations are created in the atomic ensemble and the classical bounds for *α* and for 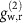 are recovered (Supplementary Note 1).

### Storage in the Rydberg ensemble

**Figure 3:**
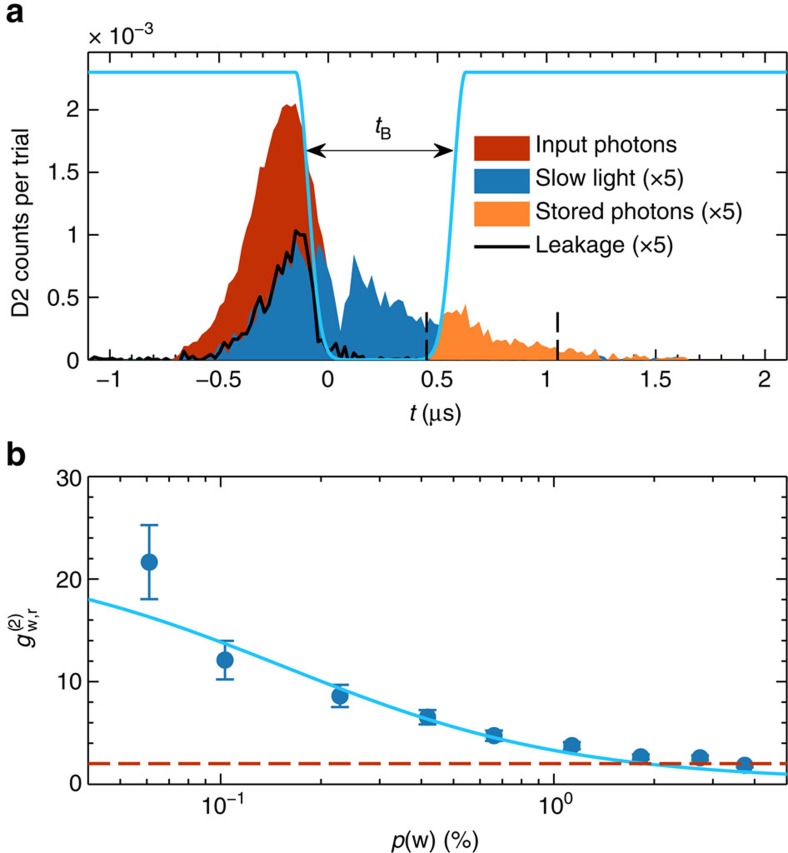
Single-photon storage sample and cross-correlation. (**a**) Example of single-photon storage for *t*_A_∼1 μs and *p*(*w*)=2.7%. Detected counts of single-photon detector D2 per trial and per temporal bin, conditioned on a detection of a write photon, as a function of the detection time *t* when no atoms are loaded in site B (red area), when the read photon is slowed by the presence of the coupling beam (that is, when the coupling beam is kept on, blue area) and when the read photon is stored and retrieved for *t*_B_=500 ns (orange area). We attribute the dip at *t*∼0 μs observable in the slow light pulse to the fast switch-off of the trailing edge of the input read photon (see refs 44, 45)). The solid black line represents a leakage of the slowed read photon due to low optical depth of the ensemble in site B. The solid light blue line is a pictorial representation of the coupling beam power. The vertical dashed lines shows the 600 ns temporal window chosen for measuring *p*(w, r_2_). In this example, the storage efficiency is *η*_B_=3.4%. We refer the reader to Supplementary Note 5 and Supplementary Fig. 4 for a description of the cross-correlation function across the slowed-down pulse. (**b**) 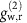 as a function of *p*(w) after storage and retrieval of the read photon for *t*_B_=500 ns. The error bars represent the propagated Poissonian error of the photon counting probabilities. The solid line is a fit with a model given in Supplementary Note 2, from which we extract the intrinsic retrieval efficiency of the DLCZ source *η*_A_=38.5%. Dashed horizontal line shows the classical bound 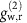
=2.

We then store the emitted single photon in a collective high-lying Rydberg atomic excitation (Fig. 3a). Keeping a fixed *t*_A_∼1 μs, we load the atoms in site B and we store the read photon as atomic coherence between states 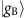 and 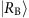 by switching off the coupling beam while the photon is propagating through the ensemble. After a storage time *t*_B_, we retrieve the stored excitation by switching the coupling beam back on. At *t*_B_=500 ns, we achieve a storage and retrieval efficiency of *η*_B_=3.4±0.4%, where *η*_B_ is defined as *η*_B_=*p*(r_2_|w)/*p*_0_(r_2_|w). We also measure 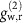
=*p*(w, r_2_)/*p*(w)*p*(r_2_) after storage and retrieval (Fig. 3b). Our data show that 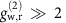 for low *p*(w) demonstrating the persistence of non-classical correlations between the write photon and the collective Rydberg atomic excitation after storage. At *t*_B_=500 ns, we explicitly violate the Cauchy–Schwarz (CS) inequality by three to four s.d.'s (Table 1), which states that a pair of classical light fields must satisfy (see ref. 36) 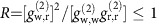, where 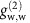 and 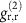 are the unheralded second-order autocorrelation functions of the write and read photon, for which a similar expression as for 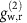 holds (Supplementary Note 1). For the same storage time, we also measured the anti-bunching parameter 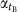 of the stored and retrieved read photon by a HBT measurement after site B and we found 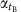
=1.2±0.2 at *p*(w)=3.98% and 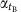
=0.0±0.35 at *p*(w)=0.59%, the latter confirming that the single-photon statistics are preserved after storage and retrieval (see Methods).

**Figure 4:**
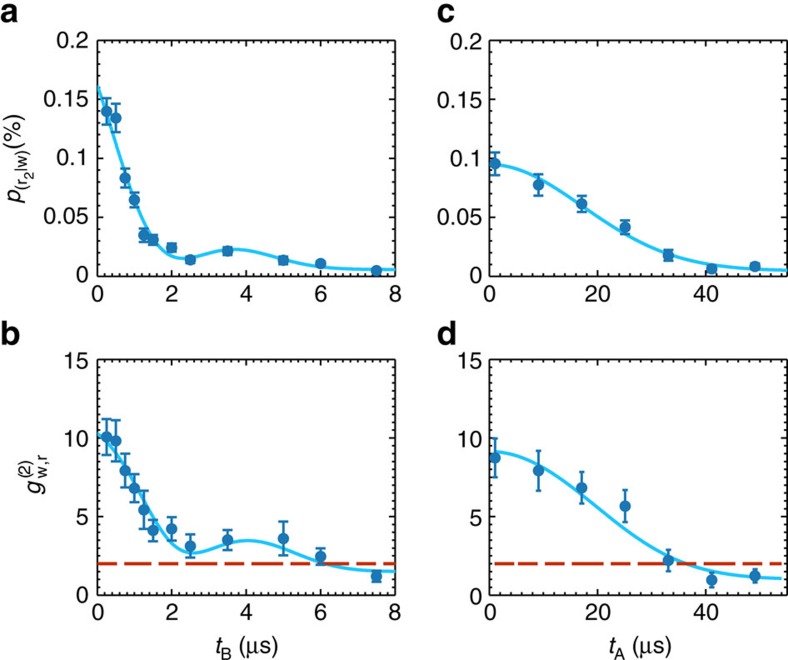
Coincidence detection probability and cross-correlation functions. Coincidence detection probability *p*(r_2_|w) and 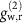 as function of *t*_B_ for *t*_A_∼1 μs (**a**,**b**) and as a function of *t*_A_ for *t*_B_=500 ns (**c**,**d**). In **a**, the measured *p*(*r*_2_|*w*) at *t*_B_=500 ns corresponds to a storage and retrieval efficiency *η*_B_=3.8±0.4%. The solid lines are a fit with the model described in the Supplementary Notes 2 and 4, from which we extract the 1/*e* decay times of *p*(r_2_|w) being 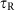
=3.3±0.3 μs and 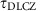
=24±2 μs for **a**,**b**, respectively. The error bars represent the propagated Poissonian error of the photon counting probabilities.

The memory capabilities of the Rydberg ensemble and of the DLCZ QM are studied in Fig. 4a) for *p*(w)=0.16±0.02%. First, we show *p*(r_2_|w) and 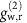 as a function of *t*_B_ (Fig. 4a,b) keeping a fixed *t*_A_∼1 *μ*s. *p*(r_2_|w), along with 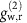, decreases when increasing the storage time, due to atomic motion and external residual fields that dephase the collective Rydberg state of equation (1). We also observe a oscillatory revival that we attribute to the hyperfine splitting Δ*F* of the Rydberg state 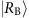 resulting in a beating of *p*(r_2_|w) with a period *T*=1/Δ*F*. The non-classical correlations between a photon and a stored Rydberg excitation are preserved up to around *t*_B_∼6 μs. Fitting *p*(r_2_|w) and 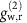 with a model shown in Supplementary Notes 2 and 4, we extract the 1/*e* decay time of the storage efficiency, 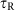
=3.3±0.3 μs as well as Δ*F*=170±16 kHz, the latter being compatible with the theoretical value of Δ*F*_theo_=182.3 kHz.

We also verify that we can generate the write and the read photon with long, controllable delay in site A, maintaining the non-classical correlation between them after storage and retrieval in site B. This result is shown in Fig. 4c,d where we change the read-out time *t*_B_ of the stored ground-state spin-wave while keeping a fixed *t*_B_=500 ns. Here the ground-state storage ensures a storage time longer than in the Rydberg state. In this case, the 1/*e* decay time is 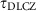
=24±2 μs and we observe non-classical correlations between the write and the stored and retrieved read photon in site B up to *t*_A_∼30 *μ*s.

### Nonlinear response of the Rydberg ensemble

**Figure 5:**
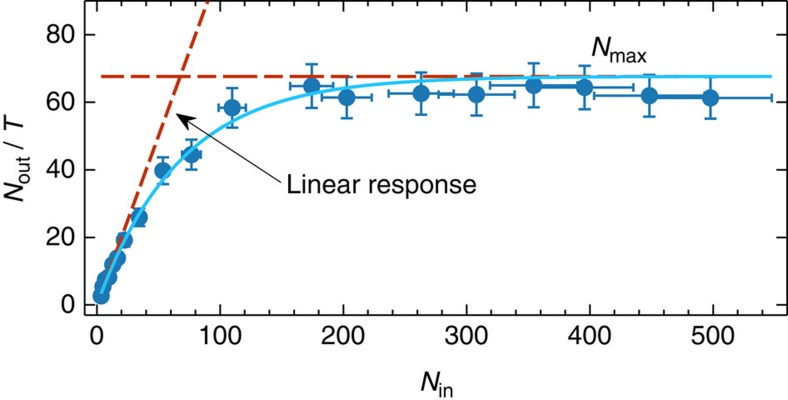
Nonlinear response of the Rydberg blockaded ensemble in site B. We store weak coherent states with a varying mean number of photon *N*_in_ into the Rydberg state 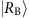 for a storage time *t*_A_=4 μs and we measure the mean number of output photon *N*_out_. We plot *N*_out_ normalized by the storage efficiency at low number of photons *T* as a function of *N*_in_. Due to Rydberg induced photon blockade, the medium can stand a maximum of *N*_max_=68±8. The solid line is a fit with the model described in the Supplementary Note 4. In this example, *T*=0.44±0.02%. The error bars are the propagated Poissonian error of the photon counting probabilities.

Finally, we prove the highly nonlinear response of the Rydberg ensemble. This is demonstrated by storing for 4 μs WCSs with varying mean number of input photon *N*_in_ and measuring the mean number of photons in the retrieved pulse after storage *N*_out_, in a way presented in ref. 28. For a linear medium, *N*_out_=*TN*_in_, where *T* is the storage efficiency, while here we show (Fig. 5) strong nonlinear dependence. Dipole–dipole interactions prevent many excitations to be stored and retrieved in the medium, which can therefore sustain no more than *N*_max_ photons. As a consequence *N*_out_ becomes 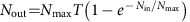
22. Our result shows (see Methods) *N*_max_=68±8, although the nonlinear dependence of *N*_out_ with respect to *N*_in_ appears at a lower number of photons. It should be noted that this result is obtained with a standard magneto-optical trap with a moderate atomic density, and that this result shows a nonlinearity six times stronger than the one reported in ref. 28. As demonstrated in refs 15, 18, 19, increasing the density of atomic ensemble with known atomic trapping techniques will allow us to achieve nonlinearity at the single-photon level, as required for applications in quantum information science.

To summarize, we have demonstrated for the first time storage and retrieval of a paired single photon on a highly nonlinear medium based on an atomic ensemble. The nonlinearity relies on highly excited Rydberg states where the capability of successfully storing a single photon is of particular importance for implementing high-fidelity quantum gates. The source is based on an emissive QM with multimode capability^42^, which is particularly suitable for quantum networking applications. Connecting this type of source with a highly nonlinear medium represents a building block for quantum networks where the entanglement can be deterministically shared over long distance by deterministic BSMs.

## Methods

### DLCZ ensemble

In site A, the measured optical depth is OD∼5 on the 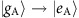 transition. A bias magnetic field *B*=110 mG along the read and write photon direction defines the quantization axes. Write and read pulses are opposite circularly polarized *σ*− and *σ*_+_, respectively. The write pulses have a Gaussian temporal shape of duration FWHM∼20 ns. The power and the temporal shape of the read pulses have been tailored to optimize the SNR of the stored and retrieved read photon which results in a Gaussian shape of FWHM∼350 ns. The angle between the write/read pulses and the write/read photon is *θ*=3.4°. From *θ* and from the Gaussian decay time 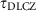 extracted from the fit shown in Fig. 4c,d, we calculate an atomic temperature of *T*_A_=77 μK (Supplementary Note 1). An optical cavity of finesse *F*=200 resonant with the write photons is used in front of detector D1 as a frequency filter in combination with a polarizing beam splitter, a quarter-wave plate and a half-wave plate that serve as polarization filtering.

### Rydberg ensemble

In site B, the measured OD on the 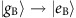 is OD∼5.5. The Rabi frequency of the coupling beam is Ω_c_=2.66±0.06 MHz, which results in a width of the EIT line of FWHM∼0.7 MHz. The magnetic field is nulled via microwave spectroscopy. The read photon and the coupling beam are focused to waists radii 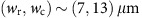, respectively. From the Gaussian decay 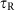, we extracted an atomic temperature of *T*_B_=38±6 μK (Supplementary Note 4). The overall detection efficiency including fibre coupling losses and efficiency of the SPD D2 is *η*_det_=15.2%

### Measurement of coincidences

To measure coincidences, we use a temporal window of 600 ns around the stored and retrieved read photon and a temporal window of 60 ns around the detected write photon. The measured value of the anti-bunching parameter after *t*_B_=500 ns, 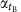
=0.00±0.35, at *p*(*w*)=0.59% corresponds to zero counts in the coincidence windows after 19 h of data acquisition.

### Single-photon Rydberg memory linewidth

The single-photon Rydberg memory linewidth is set by the width of the EIT line in combination with the single-photon bandwidth. In Supplementary Fig. 5, we show *p*(*r*_2_|*w*) after storage and retrieval of the read photon for *t*_B_=500 ns in the Rydberg state 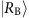, as a function of the coupling beam detuning *δ*_*c*_ with respect to the transition 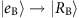. We fit the result with a Gaussian function and we extract a width of FWHM=2.38±0.09 MHz, which is the convolution of the EIT linewidth and the read photon spectral width. From the measured EIT linewidth (FWHM_EIT_=730 kHz), we find a read photon spectral width of 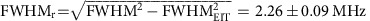. This proves that the heralded read photon can be generated in a DLCZ scheme with sub-natural linewidth in a given temporal mode, as demonstrated in ref. 41. Still, the spectral width of the read photon is slightly larger than the Fourier transform of its duration. We attribute this discrepancy to the long-term laser drift.

### Characterization of the nonlinearity

Data for Fig. 5 are taken with an increased Rabi frequency of the coupling Ω_c_=4.7±0.1 MHz, which results in a width of the EIT window of FWHM=1.3±0.04 MHz. Still the storage efficiency at low photon number *T* decreased with respect to data in Fig. 4. We attribute this decrease to an external stray electric field that fluctuates during time.

### Measurement of the cross-correlation function

As shown in Supplementary Fig. 1, we build a start-stop histogram where the start is a write photon detection and the stop is a read photon detection and we measure the number coincidence detection events in the SPDs D1 and D2, *C*_D1,D2_. We then compare *C*_D1,D2_ with the coincidences due to accidental uncorrelated detections, 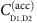. To measure *C*_D1,D2_, we count the coincidences in a 60 ns-long temporal window in D1 and in a 600 ns-long temporal window in D2. The two detection windows are temporally separated by a time *t*_A_+*t*_B_, to take into account the storage time in the two ensembles. 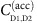 is measured by counting the coincidences between a fist write photon and a read photon detection coming from a successive uncorrelated trial. We then measure the cross-correlation via:


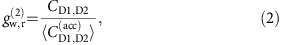


where 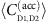 is the average number of coincidences in the extra trials, that is, second to seventh peak in Supplementary Fig. 1.

### Data availability

The data appearing in Figs 2, 3b and 4 are available in Zenodo with the identifier doi:10.5281/zenodo.165760^43^. Other data may be available upon reasonable request.

## Additional information

**How to cite this article:** Distante, E. *et al*. Storing single photons emitted by a quantum memory on a highly excited Rydberg state. *Nat. Commun*. **8**, 14072 doi: 10.1038/ncomms14072 (2017).

**Publisher's note**: Springer Nature remains neutral with regard to jurisdictional claims in published maps and institutional affiliations.

## Supplementary Material

Supplementary Information Supplementary Figures, Supplementary Notes & Supplementary References.

## Figures and Tables

**Table 1:** Reported value of the *R* parameter for the Cauchy–Schwarz inequality.

*p*(w) (%)	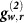	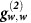	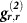	*R*
3.7	1.8±0.2	1.90±0.02	1.5±0.3	1.2±0.3
1.13	3.7±0.3	1.97±0.03	1.6±0.3	4.4±1.0
0.66	4.7±0.5	2.00±0.06	1.5±0.5	7.7±2.6

Data are taken for *t*_A_=1 μs and *t*_B_=500 ns. For low *p*(w), the CS inequality is explicitly violated.
