# Supplementary material for: Storing single photons emitted by a quantum memory on a highly excited Rydberg state
Source: Nat Commun. 2017 Jan 19;8:14072. doi: 10.1038/ncomms14072 (PMC5253638; doi:10.1038/ncomms14072)
Supplement: Supplementary Information — Supplementary Figures, Supplementary Notes & Supplementary References. [file ncomms14072-s1.pdf]

Supplementary Figures

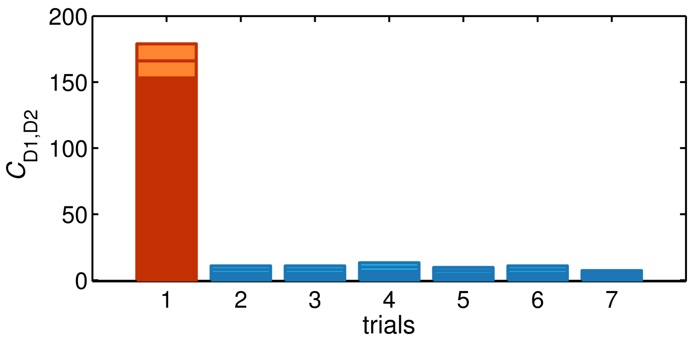

Supplementary Figure 1: Number of coincidences detection events in the SPDs D1 and D2 as a function of the number of trials. The first peak (red) represents the coincidences detection between a write and a read photon proceeding from the same readout trial $C^{(1)}_{\mathrm{D1,D2}}$, while the blue peaks are the coincidences between a write photon and a read photon proceeding from a successive uncorrelated trial. The light area in each peak represents the Poissonian measurement uncertainty.

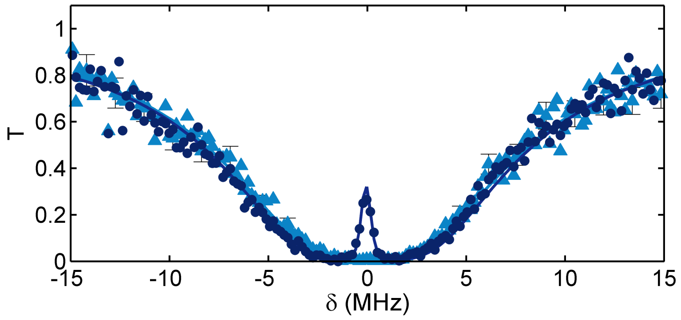

Supplementary Figure 2: Transmission of the probe weak coherent state as a function of its detuning δ when the coupling beam is off (light blue triangles) and when it is on (blue circles). The solids lines of the respective colours represent a fit to the data with the model described in [1] from we extract $\mathrm{OD} = 5.4 \pm 0.1$, $\Omega_{\mathrm{c}} = 2.66 \pm 0.06\,\mathrm{MHz}$, full width half maximum of the EIT peak $\mathrm{FWHM} = 0.73 \pm 0.03\,\mathrm{MHz}$ peak transparency $T_0 = 32.1\%$ and dephasing rate $\gamma_{\mathrm{gR}} = 0.29 \pm 0.03\,\mathrm{MHz}$.

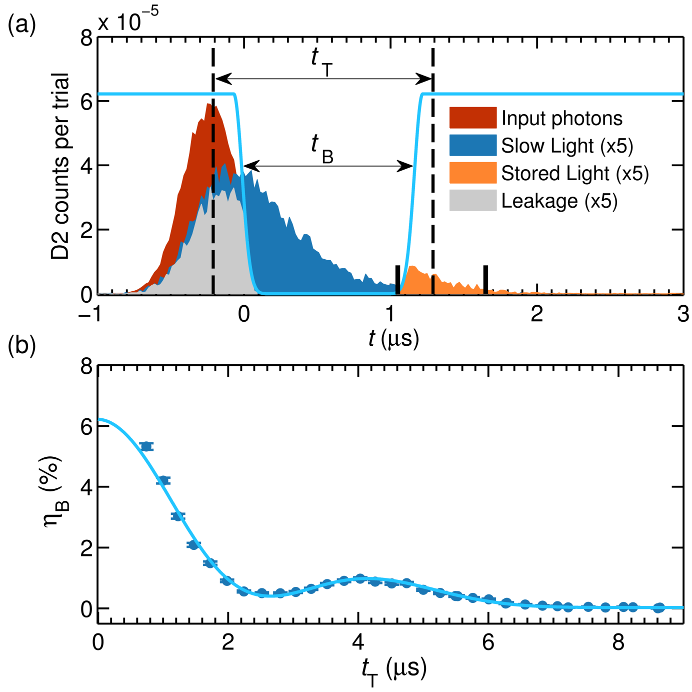

Supplementary Figure 3: (a)Example of storage and slow light of a weak coherent state. Here we show: the input probe pulse measured when the atomic ensemble is not loaded (red), slowed pulse transmitted through the cloud when the coupling beam remains on (blue), the stored and retrieved pulse (orange) and the leakage due to low OD (grey). The ratio between the area of the slowed pulse and the input pulse is $\eta_{\text{slow}} = A_{\text{slow}}/A_{\text{in}} = 23\,\%$. The vertical dashed line indicate the centre of mass of the input and of the retrieved pulse respectively which we use to measure t_{T} (see text). We calculate the storage efficiency η_{B} considering a time window of 600 ns, indicated by the black vertical lines. In this example, $\eta_{\text{B}} = 2.2\,\%$ for $t_{\text{B}} = 1\,\mu$s corresponding to $t_{\text{T}} \sim 1.47\,\mu$s. The solid blue line represents the intensity of the coupling beam. (b) Storage efficiency as a function of t_{T} together with a fit with the equation (17). From the fit shown we extract a coherence time $\tau_{\text{R}} = 3.34 \pm 0.02\,$ns and a frequency separation of the hyperfine states $\Delta F = 194 \pm 4\,$kHz.

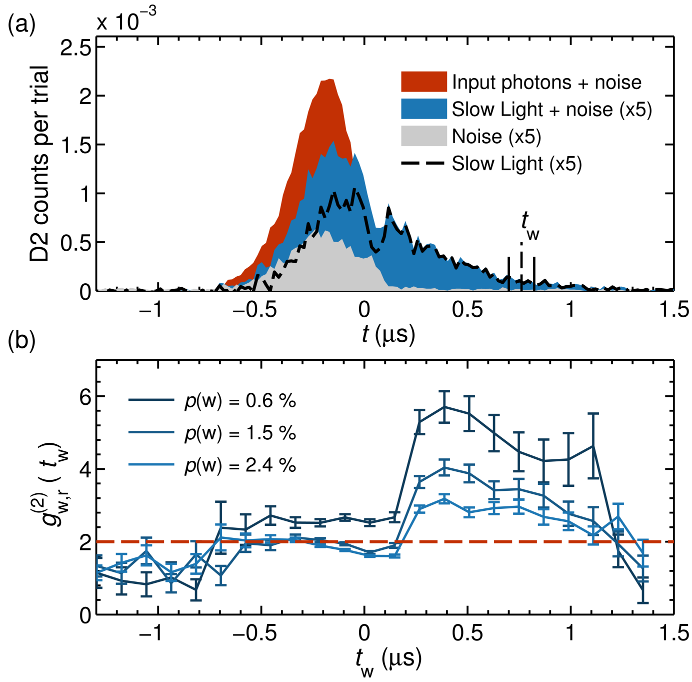

Supplementary Figure 4: (a) Count rate on the SPD D2 when no atoms are loaded in site B (red), when the atoms are loaded and the pulse is slowed down (blue), when the atoms are loaded and the coupling beam is switched off, representing the unwanted noise on the $|5S_{1/2}, F=1\rangle \leftrightarrow |5P_{3/2}, F=2\rangle$ transition (grey). The dashed line is the noise subtracted slowed read pulse. The dip in the slowed pulse that is observable at $t \sim 0\,\mu$s results from the fast switch-off of the trailing edge of the input photon (see [2, 3]). The two solid vertical lines show the $\sim 123\,$ns time window gate centred at t_{w} (marked by the dashed vertical line) that we use to measure the cross-correlation function in (b). (b) Cross-correlation function as a function of t_{w}. The horizontal line represents the classical bound $g^{(2)}_{\text{w,r}} = 2$.

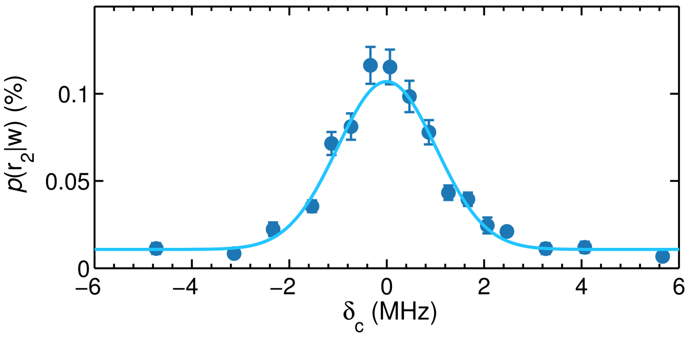

Supplementary Figure 5: Coincidence detection probability $p(r_2|w)$ as a function of the the coupling beam detuning δ_c after storing the read photon for t_R = 500 ns. The fit with the function $Ae^{-\delta_c^2/2\sigma^2}$ (solid line) gives a width $\sigma = 1.01 \pm 0.04$ (FWHM $= 2.38 \pm 0.09$ MHz). The error bars are the Poissonian error of the photon counting statistics.

Supplementary Notes

Supplementary Note 1: Theoretical background of a DLCZ quantum memory

In this section we review the basics of a DLCZ quantum memory. In site A, N_{A} atoms are prepared in the ground state, and their collective state is written as $|G\rangle = \left|g_{\mathrm{A}_1}...g_{\mathrm{A}_i}...g_{\mathrm{A}_{N_{\mathrm{A}}}}\right\rangle$. A write pulse generates with a probability p at least a write photon in a given spatial mode by Raman scattering, transferring the atoms in the ground state $|s_{\mathrm{A}}\rangle$. The atom-light system can be described by the two-mode squeezed state:

$$|\psi_A\rangle = \sqrt{1-p}\sum_{n=1}^{\infty} p^{n/2}\frac{\left(a_{\mathrm{w}}^{\dagger}S^{\dagger}\right)^n}{n!}|0_{\mathrm{w}}\rangle|G\rangle\,. \tag{1}$$

Here the subscript w indicates the write photon mode, $a_{\mathrm{w}}^{\dagger}$ and $S^{\dagger}$ are the creation operators of the write photon and of the collective state of the atoms, explicitly:

$$S^{\dagger} = \frac{1}{\sqrt{N_A}}\sum_{j=1}^{N_A} e^{-i(\mathbf{k}_{\mathrm{W}}-\mathbf{k}_{\mathrm{w}})\cdot\mathbf{r}_j}\left|s_{\mathrm{A}_j}\right\rangle\left\langle g_{\mathrm{A}_j}\right|, \tag{2}$$

where $\mathbf{k}_{\mathrm{W}}$ and $\mathbf{k}_{\mathrm{w}}$ are the wavevectors of the write pulse and the write photon, and $\mathbf{r}_j$ is the position of the $j^{\mathrm{th}}-$atom. From equation (1), one can see that for low p the atoms share a single, collective spin excitation (also called *spin-wave*)

After a storage time t_{A} the atomic excitation is mapped deterministically into a read photon via Raman scattering by mean of an intense read pulse. Defining the read pulse wavevector as $\mathbf{k}_{\mathrm{R}}$, the read photon spatial-mode will be given by the phase-matching condition $\mathbf{k}_{\mathrm{r}} = \mathbf{k}_{\mathrm{W}} + \mathbf{k}_{\mathrm{R}} - \mathbf{k}_{\mathrm{w}}$, while the temporal mode will depend on the temporal shape of the read pulse [4]. The pair state of the write and read photon then reads:

$$|\varphi\rangle = \sqrt{1-p}(|0_{\mathrm{w}}0_{\mathrm{r}}\rangle + \sqrt{p}\,|1_{\mathrm{w}}1_{\mathrm{r}}\rangle + p\,|2_{\mathrm{w}}2_{\mathrm{r}}\rangle + O(p^{3/2})), \tag{3}$$

where now the subscript r stands for the read photon mode. From equation (3), it is evident that for low p a successful detection of a write photon project the read photon into a single photon state.

The write and read photon statistics can be measured by their second-order auto-correlation function $g^{(2)}_{\mathrm{w,w}}$ and $g^{(2)}_{\mathrm{r,r}}$ via HBT measurement. Taking as example the write photon mode (similar expression holds for the read photon). we denote w_1 and w_2 the write photon mode after passing through a balance beam splitter, $p(\mathrm{w}_i)$ the probability to detect a single photon in the w_i photon mode, and $p(\mathrm{w}_1, \mathrm{w}_2)$ the probability of a coincidence detection of photons between the modes w_1 and w_2, the auto-correlation function reads:

$$g^{(2)}_{\mathrm{w,w}} = \frac{p(\mathrm{w}_1,\mathrm{w}_2)}{p(\mathrm{w}_1)p(\mathrm{w}_2)} = \frac{\langle a^{\dagger}_{\mathrm{w}_1}a^{\dagger}_{\mathrm{w}_2}a_{\mathrm{w}_2}a_{\mathrm{w}_1}\rangle}{\langle a^{\dagger}_{\mathrm{w}_1}a_{\mathrm{w}_1}\rangle\langle a^{\dagger}_{\mathrm{w}_2}a_{\mathrm{w}_2}\rangle}. \tag{4}$$

From Eq. (4) one finds $g^{(2)}_{\mathrm{w,w}} = g^{(2)}_{r,r} = 2$ for the two-mode squeezed state given by equation (3). This means that taken individually the write and the read photon are in a pure thermal state. Stray light and coherent leakage noise diminishes the auto-correlation function thus one finds $1 \leq g^{(2)}_{\mathrm{w,w}} \leq 2$ and $1 \leq g^{(2)}_{\mathrm{r,r}} \leq 2$, as also shown by our measurement (see Table I in the main text).

On the contrary, after a successful detection of a write photon, for low p the read photon mode is projected onto a single photon states, showing anti-bunching in a HBT measurement. That means that its *heralded* auto-correlation function, also called the anti-bunching parameter $\alpha = g^{(2)}_{(r,r|w)}$, must be $\alpha \ll 1$. Explicitly:

$$\alpha = \frac{p(\mathrm{r}_1,\mathrm{r}_2|\mathrm{w})}{p(\mathrm{r}_1|\mathrm{w})p(\mathrm{r}_2|\mathrm{w})} = \frac{\langle a^{\dagger}_{\mathrm{w}}a^{\dagger}_{\mathrm{r}_1}a^{\dagger}_{\mathrm{r}_2}a_{\mathrm{r}_2}a_{\mathrm{r}_1}a_{\mathrm{w}}\rangle\langle a^{\dagger}_{\mathrm{w}}a_{\mathrm{w}}\rangle}{\langle a^{\dagger}_{\mathrm{w}}a^{\dagger}_{\mathrm{r}_1}a_{\mathrm{r}_1}a_{\mathrm{w}}\rangle\langle a^{\dagger}_{\mathrm{w}}a^{\dagger}_{\mathrm{r}_2}a_{\mathrm{r}_2}a_{\mathrm{w}}\rangle}, \tag{5}$$

where it can be shown that

$$\alpha = 2p\frac{(2+p)}{(1+p)^2}, \tag{6}$$

and $\alpha \underset{p\to 0}{\longrightarrow} 4p$.

The correlations between the write and the read photon can be measured by the second-order cross-correlation function $g^{(2)}_{\mathrm{w,r}}$, which as a function of p is:

$$g^{(2)}_{\mathrm{w,r}} = \frac{\langle a^{\dagger}_{\mathrm{w}}a^{\dagger}_{\mathrm{r}}a_{\mathrm{r}}a_{\mathrm{w}}\rangle}{\langle a^{\dagger}_{\mathrm{w}}a_{\mathrm{w}}\rangle\langle a^{\dagger}_{\mathrm{r}}a_{\mathrm{r}}\rangle} = 1 + \frac{1}{p} \tag{7}$$

from which it is clear that for high-quality single photon (i.e. $\alpha \sim 0$) one gets $g^{(2)}_{\mathrm{w,r}} \gg 1$.

Non-classical correlations between the write and the read photons arise when $g^{(2)}_{\mathrm{w,r}} > 2$. This can be inferred from the Cauchy-Schwartz inequality (CS) which states that two classical light fields fulfil $R = \left[g^{(2)}_{\mathrm{w,r}}\right]^2/g^{(2)}_{\mathrm{w,w}}g^{(2)}_{\mathrm{r,r}} \leq 1$. Since in our situation $1 \leq g^{(2)}_{\mathrm{w,w}} \leq 2$ and $1 \leq g^{(2)}_{\mathrm{r,r}} \leq 2$ for the write and the read photon, it is easy to see that $g^{(2)}_{\mathrm{w,r}} > 2$ violates the CS inequality.

Supplementary Note 2 Description of the photon emission from the DLCZ photon source including noise

Here we show the theoretical model that we use to fit data in Fig. 2(a,b), 3b, 4(a,b,c,d) in the main text. Following a similar procedure as in [4], we start from the ideal description of a DLCZ QM and we include the noise contributions given by spontaneous emission, stray light fields and dark-counts of the SPDs.

As explained in detail above, the write process creates pairs consisting of a write photon and a spin-wave in a probabilistic way. These spin-waves can be later converted with a high probability into read photons emitted in a particular transition and direction, due to collective interference. The quantities that we measure in this work depend on how directional is the emission of these read photons. The ratio between directional and random emission depends, among other factors, on the preservation of the spin-wave coherence or the optical depth of the atomic ensemble.

The ratio of directional emission can be characterized by the intrinsic retrieval efficiency η_{A}. The random emission is proportional the total number of atoms in the $|s_{\mathrm{A}}\rangle$ ground state, the read photon spatial mode solid angle and to global branching ratio corresponding to the detectable transitions, p_{SE}. Following a similar procedure as in [4], we can write all the photon detection probabilities as:

$$p(\mathrm{w}) = p\eta_{\mathrm{w}} + p_{n\mathrm{w}}, \tag{8}$$

$$\begin{aligned} p(\mathrm{r}) &= p(\mathrm{r})^{dir} + p(\mathrm{r})^{\mathrm{rand}} = \\ &= p\eta_{\mathrm{A}}\eta_{\mathrm{r}} + p(1-\eta_{\mathrm{A}})p_{\mathrm{SE}}\eta_{\mathrm{r}} + p_{n\mathrm{r}}, \end{aligned} \tag{9}$$

$$\begin{aligned} p(\mathrm{w},\mathrm{r}) &= p(\mathrm{w},\mathrm{r})^{dir} + p(\mathrm{w})p(\mathrm{r})^{\mathrm{rand}} = \\ &= p(\mathrm{w})\eta_{\mathrm{A}}\eta_{\mathrm{r}} + p(\mathrm{w})p(1-\eta_{\mathrm{A}})p_{\mathrm{SE}}\eta_{\mathrm{r}} + p(\mathrm{w})p_{n\mathrm{r}}, \end{aligned} \tag{10}$$

where p is the probability to create a spin-wave together with a write photon in the coupled spatial mode, η_{w} is the write photon total detection efficiency, η_{r} is the total transmission of the read photon after leaving the atomic ensemble (including the Rydberg storage efficiency η_{B}) and $p_{n\mathrm{w}}$ ($p_{n\mathrm{r}}$) is the probability to detect a background count (including stray light and SPD dark-counts) in the write (read) photonic mode. To fit data of Fig. 2a of the main text we used equation (6) substituting p with $c_1 p(\mathrm{w}) + c_2$. The terms c_1 and c_2 are used to include the noise on the detectors D1, D3 and D4.

From Eqs. (8, 9, 10) we compute the function:

$$g^{(2)}_{\mathrm{w,r}} = \frac{p(\mathrm{w},\mathrm{r})}{p(\mathrm{w})p(\mathrm{r})}, \tag{11}$$

which is used to fit the data of Fig. 2b and 3b in the main text. The free parameters are η_{A}, p_{SE}, and p_{nr}. In our experiment, the SPD of the write photon is gated for a very short time $t_{\mathrm{w}}^{gate} \sim 60\,\mathrm{ns}$, therefore we measured $p_{nw} \ll p(w)$.

To fit data in Fig. 4(a,b) of the main text, a storage time dependence of the Rydberg storage efficiency has to be included. To do so, we considered a time-dependent η_r, where the time dependence follows equation (17) (see next section). Similarly, for the data in Figures 4(c,d) the delay between the write and read pulses is changed and the decoherence of the spin-wave has to be considered. Decoherence decreases the directionality of the read photon emission. In our experiment this is mainly due to the motion of the atoms and it gives a Gaussian decay of the intrinsic retrieval efficiency $\eta_{\mathrm{A}}(t) = \eta_{\mathrm{A}} e^{-t^2/\tau^2_{\mathrm{DLCZ}}}$ [5], being $\tau_{\mathrm{DLCZ}} = \sqrt{m/(k_B T \Delta k^2)}$, where m is the atomic mass, k_{B} is Boltzmann's constant, T is the atomic temperature and $\Delta k = |\mathbf{k}_{\mathrm{W}} - \mathbf{k}_{\mathrm{w}}|$ is the difference between the write pulse and write photon wavevectors.

Supplementary Note 3 Theoretical background of electromagnetically induced transparency

Here we review briefly the basics of the electromagnetically induced transparency (EIT) in site B. The atomic cloud in site B can be described as a system of three level atoms, the levels being: the ground state $|g_{\mathrm{R}}\rangle = |5S_{1/2}, F=2\rangle$, the excited state $|e_{\mathrm{B}}\rangle = |5P_{3/2}, F=2\rangle$ and the Rydberg state $|R_{\mathrm{B}}\rangle = |60S_{1/2}\rangle$. We probe the atomic cloud by measuring the transmission through the cloud of a weak coherent field ($\mathcal{E}$) detuned by δ with respect to the $|g_{\mathrm{B}}\rangle \to |e_{\mathrm{B}}\rangle$ transition. When the detuning approaches the resonant condition, i.e. $\delta = 0$, the atoms absorb the probe field, the transmission drops to its minimum value $T(\delta = 0) = e^{-\mathrm{OD}}$, where OD is the Optical Depth of the cloud for this transition. By fitting the data of the transmission as a function of δ we extract the OD of the cloud, being $\mathrm{OD} = 5.41 \pm 0.14$.

A strong coupling beam with Rabi frequency Ω_{c} resonant with the $|e_{\mathrm{B}}\rangle \to |R_{\mathrm{B}}\rangle$ transition creates the conditions for EIT opening up a window of transparency in the transmission spectrum of the probe field around $\delta = 0$. We extract Ω_{c} as well as the dephasing rate γ_{gR} of the $|g_{\mathrm{B}}\rangle \to |R_{\mathrm{B}}\rangle$ transition by fitting the transmission as a function of δ with (see [1]):

$$T = \exp\{-k_{\mathrm{p}}\ell \; Im\,[\chi(\delta)]\}, \tag{12}$$

$$\chi(\delta) = \mathrm{OD}\frac{\Gamma}{2k_{\mathrm{p}}\ell}\left(\frac{\delta + i\gamma_{\mathrm{gR}}}{(\Gamma/2 - i\delta)(\gamma_{\mathrm{gR}} - i\delta) + (\Omega_{\mathrm{c}}/2)^2}\right), \tag{13}$$

where Γ is the linewidth of the $|g_{\rm B}\rangle \rightarrow |e_{\rm B}\rangle$ transition, k_p is the probe beam wavenumber and ℓ is the atomic cloud length. An example of the transmission with and without the coupling beam is shown in Supplementary Figure 2. The dephasing $\gamma_{\rm gR}$ include the lasers linewidth, the lifetime of the $|R_{\rm B}\rangle$ state as well as the broadening induced by spurious external fields. $\gamma_{\rm gR}$ in combination with $\Omega_{\rm c}$ sets the height and the width of the EIT transparency window. The height of the peak is further limited by imperfect mode-matching between the probe beam and the coupling beam.

Supplementary Note 4 Light storage with EIT

When $\delta = 0$, the coupling beam converts the probe field into a slowly propagating *dark-state polariton* (DSP), described by the field operator $\phi(z,t)$. A DSP is a coherent superposition of the probe electric field $\mathcal{E}(z,t)$ and the atomic coherence between the ground and - in our case - the Rydberg state, $\sigma_{\rm g,R}(z,t)$. For a medium with density ρ the field of a DSP writes [6]:

$$\boldsymbol{\phi}(\mathbf{z},t) = \boldsymbol{\mathcal{E}}(\mathbf{z},t)\cos(\theta) - \sqrt{\rho}\,\boldsymbol{\sigma}_{\rm g,R}(\mathbf{z},t)e^{-\boldsymbol{\Delta}\mathbf{k}\cdot\mathbf{z}}\sin(\theta) \tag{14}$$

where the mixing angle θ is related to the group index $n_{\rm gr}$ of the medium following $\tan^2(\theta) = n_{\rm gr}$ and $\boldsymbol{\Delta}\mathbf{k} = \mathbf{k}_{\rm c} + \mathbf{k}_{\rm p}$, where $\mathbf{k}_{\rm c}$ ($\mathbf{k}_{\rm p}$) is the wavevector of the coupling (probe) field.

The group index depends on $\Omega_{\rm c}$ and on the OD of the $|g_{\rm B}\rangle \rightarrow |e_{\rm B}\rangle$ transition. In particular $n_{\rm gr} \sim {\rm OD}/\Omega_{\rm c}^2$, as consequence a DSPs propagates at a reduced group velocity $v_{\rm gr} = c/n_{\rm gr}$. In the limit $\Omega_{\rm c} \rightarrow 0$, the group index $n_{\rm gr}$ goes to infinity and the group velocity of the polariton $v_{\rm gr}$ is reduced to zero. In this condition $\theta \rightarrow \pi/2$, therefore $\cos(\theta) \rightarrow 0$ and $\sin(\theta) \rightarrow 1$. This means that by switching off the coupling field it is possible to convert all the probe light into stationary atomic coherence, effectively storing the probe pulse as atomic coherence. In this situation, the state of the atomic ensemble can be written as a collective Rydberg atomic excitation

$$|\psi_{\rm B}\rangle = \frac{1}{\sqrt{N_{\rm B}}}\sum_{i=1}^{N_{\rm B}} e^{-i(\mathbf{k}_{\rm c}+\mathbf{k}_{\rm p})\mathbf{r}_i}\left|g_{{\rm B}_1}...R_{{\rm B}_i}...g_{{\rm B}_{N_{\rm B}}}\right\rangle. \tag{15}$$

By switching the coupling beam back on, the light component of the polariton is restored and the pulse is retrieved out of the medium. The storage process is limited by low OD, by finite transparency at the centre of the EIT feature and by imperfect frequency bandwidth matching between the probe pulse and the narrow band EIT window. In particular, at low OD the probe pulse is not fully compressed inside the atomic ensemble, resulting in a light leakage eventually reducing the storage efficiency. In our case OD = 5.4 which gives a leaked part equal to 42 % of the transmitted slowed pulse (see Supplementary Figure 3).

The storage efficiency decreases at longer storage time due to the dephasing of the collective Rydberg atomic state described by equation (15). The main sources of dephasing are atomic motion given by finite temperature of the cloud, as well as external stray fields. In our experiment, we observe a Gaussian decay $\eta_{\rm B}(t_{\rm T}) = \eta_0 e^{-t_{\rm T}^2/\tau_{\rm R}^2}$, where now $t_{\rm T} = t_{\rm B} + t_{\rm OFF}$ is the total time that an input light pulse takes to cross the atomic sample. $t_{\rm OFF}$ is a time-offset that takes into account the delay time $\delta t = v_{\rm gr}/\ell$ that results from the reduced group velocity of the DSP as well as the temporal profile of the coupling beam. In the present letter, we measure $t_{\rm T}$ as the difference between the centre-of-mass of the stored and retrieved pulse and the input pulse see Supplementary Figure 3. By defining $f_{\rm in}(t)$ and $f_{\rm out}(t)$ the temporal shape of the input pulse and of the stored and retrieved pulse respectively, $t_{\rm T}$ reads:

$$t_{\rm T} = \frac{\int f_{\rm out}(t)\,t\,dt}{\int f_{\rm out}(t)\,dt} - \frac{\int f_{\rm in}(t)\,t\,dt}{\int f_{\rm in}(t)\,dt} \tag{16}$$

To fully understand data in Figures 4(a,b) of the main text and in Supplementary Figure 3, one must include the hyper-fine structure of the Rydberg state, $|R_{\rm B}\rangle = \left|60S_{1/2}\right\rangle$, which is composed of the two hyper-fine states $|R_{{\rm B},\,F=1}\rangle = \left|60S_{1/2}, F=1\right\rangle$ and $|R_{{\rm B},\,F=2}\rangle = \left|60S_{1/2}, F=2\right\rangle$ separated by $\Delta F_{\rm theo} = 182.1\,{\rm kHz}$. Due to our laser linewidth, we cannot resolve these two states in our EIT spectroscopy, as a consequence the probe is stored in a coherent superposition of the $|R_{{\rm B},\,F=1}\rangle$ and the $|R_{{\rm B},\,F=2}\rangle$. The energy difference between these states results in a different phase evolution and the initial phase difference is recovered every $T = 1/\Delta F_{\rm theo}$. This produces the oscillations of the storage efficiency as a function of time which then reads:

$$\eta_{\rm B}(t_{\rm B}) = \eta_0 e^{-t_{\rm T}^2/\tau_{\rm R}^2}\left|p_{F=1} + (1-p_{F=1})e^{-2\pi{\rm i}\Delta F t_{\rm T}}\right|^2, \tag{17}$$

where $p_{F=1}$ is the probability to excite the $|R_{{\rm B},\,F=1}\rangle$ state. The decay time $\tau_{\rm R}$ can be used to extract an upper bound of the atomic temperature. Considering the atomic motion as the only source of dephasing then the coherence time is $\tau_{\rm R} = \sqrt{m/(k_{\rm B}T\Delta k^2)}$ where T is the temperature of the atoms.

Supplementary Note 5 Time-dependent slow-light cross-correlation function

In this section we will show the cross-correlation function between the write and the read photon when the read photon undergoes Rydberg EIT in site B.

To understand our data (shown in Supplementary Figure 4) it is crucial to analyse the frequency component of the noise carried by the input read photon. During the reading process in site A, a spontaneous emitted photon can be

generated either in the $|5S_{1/2}, F=1\rangle \leftrightarrow |5P_{3/2}, F=2\rangle$ or in the $|5S_{1/2}, F=2\rangle \leftrightarrow |5P_{3/2}, F=2\rangle$ transitions. This is mainly due to imperfect optical pumping during the initialization of the DLCZ memory which let some unwanted population in the $|5S_{1/2}, F=1\rangle$ state. Such spontaneous emitted photons are not correlated with the write photon, therefore act as a source of noise, eventually limiting the $g^{(2)}_{\mathrm{w,r}}$.

When the read photon is stored as a collective Rydberg state, the Rydberg EIT memory acts as a beneficial frequency noise filter. In this case, only a light pulse resonant with the $|5S_{1/2}, F=2\rangle \rightarrow |5P_{3/2}, F=2\rangle$ transition can be stored and retrieved. Since the ensemble in site B is prepared in the $|5S_{1/2}, F=2\rangle$ state, an out-of-resonant spontaneous emitted photon would not interact with the atoms, leaving the ensemble in the same temporal mode as the input read photon. In general, any out of resonance noise of the input read photon can not be seen in the time window of the retrieved pulse.

This is not the case in a slow-light experiment. In Supplementary Figure 4a we show the input pulse, the noise on the $|5S_{1/2}, F=1\rangle \rightarrow |5P_{3/2}, F=2\rangle$ (measured by loading the ensemble in site B and not performing EIT) together with the slow read photon with and without noise subtraction. As one can see, the slowed pulse and the input read pulse are not temporally separated. As a result, measuring the $g^{(2)}_{\mathrm{w,r}}$ of the write and read slowed pulse using as a coincidence temporal window the whole duration of the slowed pulse would lead to $g^{(2)}_{\mathrm{w,r}} < 2$, since it would include all the out-of-resonance frequency noise components. To show this effect, we measured the $g^{(2)}_{\mathrm{w,r}}$ as a function of the time t_{w} at which we centered a 123 ns coincidence detection window. The result is plotted in Supplementary Figure 4b, where we show the cross-correlation function for three different values of $p(w)$. As one can see, in the region of time where the slowed pulse and the noise are present we found $g^{(2)}_{\mathrm{w,r}} \sim 2$. This is a combination of the uncorrelated noise, for which we expect $g^{(2)}_{\mathrm{w,r}} \sim 1$ and the non-classical correlated slowed read pulse, for which $g^{(2)}_{\mathrm{w,r}} > 2$. At longer time, while the noise is not present anymore, not being slowed down, we still have the signal of the slowed read pulse, therefore the non-classical correlations are recovered.

Supplementary References

[1] Xiao, M., *et al.* Measurement of Dispersive Properties of Electromagnetically Induced Transparency in Rubidium Atoms. *Phys. Rev. Lett.* **74**, 666–669 (1995).

[2] Xiao, M., *et al.* Optical Precursors with Electromagnetically Induced Transparency in Cold Atoms. *Phys. Rev. Lett.* **103**, 093602 (2009).

[3] Zhang, M.,*et al.* Preparation and storage of frequency-uncorrelated entangled photons from cavity-enhanced spontaneous parametric downconversion *Nat Photon* **5**, 628–632 (2001).

[4] Farrera, P. *et al.* Generation of single photons with highly tunable wave shape from a cold atomic quantum memory. *Preprint at* https://arxiv.org/abs/1601.07142. (2016).

[5] Albrecht, B., Farrera, P., Heinze, G., Cristiani, M. & de Riedmatten, H. Controlled rephasing of single collective spin excitations in a cold atomic quantum memory. *Phys. Rev. Lett.* **115**, 160501– (2015).

[6] Fleischhauer, M. & Lukin, M. D. Quantum memory for photons: Dark-state polaritons. *Phys. Rev. A* **6**, 022314 (2002).
